# WEE1 Inhibition Augments CDC7 (DDK) Inhibitor–induced Cell Death in Ewing Sarcoma by Forcing Premature Mitotic Entry and Mitotic Catastrophe

**DOI:** 10.1158/2767-9764.CRC-22-0130

**Published:** 2022-06-20

**Authors:** Jeffrey C. Martin, Jennie R. Sims, Ajay Gupta, Andrei V. Bakin, Joyce Ellen Ohm

**Affiliations:** 1 Department of Cancer Genetics and Genomics, Roswell Park Comprehensive Cancer Center, Buffalo, New York.; 2 Division of Pediatric Oncology, Roswell Park Comprehensive Cancer Center, Department of Pediatrics, University at Buffalo Jacobs School of Medicine and Biomedical Sciences, Buffalo, New York.

## Abstract

Ewing sarcoma is an aggressive childhood cancer for which treatment options remain limited and toxic. There is an urgent need for the identification of novel therapeutic strategies. Our group has recently shown that Ewing cells rely on the S-phase kinase cell division cycle 7 (CDC7) DBF4-dependent kinase (DDK) to maintain replication rates and cell viability and that DDK inhibition causes an increase in the phosphorylation of CDK1 and a significant delay in mitotic entry. Here, we expand on our previous findings and show that DDK inhibitor–induced mitotic entry delay is dependent upon WEE1 kinase. Specifically, WEE1 phosphorylates CDK1 and prevents mitotic entry upon DDK inhibition due to the presence of underreplicated DNA, potentially limiting the cytotoxic effects of DDK inhibition. To overcome this, we combined the inhibition of DDK with the inhibition of WEE1 and found that this results in elevated levels of premature mitotic entry, mitotic catastrophe, and apoptosis. Importantly, we have found that DDK and WEE1 inhibitors display a synergistic relationship with regards to reducing cell viability of Ewing sarcoma cells. Interestingly, the cytotoxic nature of this combination can be suppressed by the inhibition of CDK1 or microtubule polymerization, indicating that mitotic progression is required to elicit the cytotoxic effects. This is the first study to display the potential of utilizing the combined inhibition of DDK and WEE1 for the treatment of cancer. We believe this will offer a potential therapeutic strategy for the treatment of Ewing sarcoma as well as other tumor types that display sensitivity to DDK inhibitors.

**Significance:**

Ewing sarcoma is an aggressive pediatric cancer for which chemotherapy is very intense and often results in acute toxicities. Here, we have found that the combined inhibition of CDC7 kinase (DDK) and WEE1 kinase forces high levels of mitotic errors and synergistic cell death, specifically in Ewing sarcoma cells. This combination has the potential to provide a highly efficacious and minimally toxic treatment strategy for patients with Ewing sarcoma.

## Introduction

Ewing sarcoma is an aggressive childhood malignancy that typically presents in the bone (1). Over the past several decades, many of the advances in treatment have come in the form of technological progress, such as the development of novel prosthetics that have enabled an expansion of the number of patients that qualify for surgical resection of primary tumors (2–4). Significant advancements to chemotherapy regimens have been incremental, with most changes affecting treatment intervals and durations (5). Although many have been made, most of the molecular insights into the etiology of Ewing sarcoma have failed to manifest as clinical impacts (1). Frontline chemotherapy consists mainly of nonspecific and toxic alkylating agents and topoisomerase inhibitors. For these reasons, side effects from systemic therapy are often consequential and long lasting (6). There is a clear need for the development of novel therapeutics that can improve patient outcome while limiting treatment toxicity.

While the molecular underpinnings of Ewing sarcoma are complex and largely still under investigation, the main genetic driver has been well known for decades (7). About 95% of Ewing tumors harbor a genetic translocation between the RNA-binding protein, EWSR1, and the ETS transcription factor, FLI1 (8). The resulting fusion protein, termed EWS-FLI1, acts as an aberrant transcription factor, capable of transforming the epigenome (9, 10) and transcriptome (11) toward the malignant phenotype. Ewing sarcoma cell viability is reliant upon the presence and function of the fusion protein, highlighting its importance (12). Therefore, the direct targeting of the fusion protein poses a viable treatment option. However, the complexity of EWS-FLI1 structure and function has resulted in efforts to develop small-molecule inhibitors, or other means of inhibition, being largely unsuccessful (13). Therefore, many research groups have shifted their focus toward understanding the downstream effects of the fusion protein, whether they be primary [i.e., discovery of EWS-FLI1 target genes (11)] or secondary [i.e., discovery of exploitable adverse side effects of the fusion function (14)].

A large body of work has revealed a unique effect of the fusion function that poses a potentially lethal molecular vulnerability, replication stress (RS; ref. 14). RS refers to anything that hinders the progression of an active replisome during DNA replication (15). For example, protein barriers such as transcription complexes (16), large regions of heterochromatin (17), or aberrant frequencies of noncanonical DNA secondary structures, like R-loops (18, 19) and G-quadraplexes (20–22), all pose impediments to fork progression. Most tumors harbor some degree of RS as it is a common outcome of the oncogenic process (23, 24).

Upon its discovery, researchers understood RS to pose a vulnerability within tumors and began investigating avenues to target it (25, 26). Targeted therapy aimed at the exploitation of RS has come primarily in the form of small-molecule inhibitors of the enzymes involved in the RS response (RSR), mainly ATR, CHK1, and PARP1 (23, 26). The initial evidence for the presence of RS within Ewing tumors was the fact that they display sensitivity to several RSR kinases including ATR and PARP, as well as others such as ribonucleotide reductase (14). Recently, a group showed that an increase in RNAPII-mediated transcription results in R-loop–associated replication fork stalling, shedding light on the source of the RS (27).

Cell division cycle 7 (CDC7) otherwise known as DBF4-dependent kinase (DDK) is an S-phase kinase that, in conjunction with CDK2, works to initiate replication from replication origins through its direct phosphorylation of the MCM2-7 helicase complex (28–32). DDK activity is acutely malleable, and the enzyme is closely monitored throughout S-phase (33, 34). Its function depends upon the nuclear environment and is capable of promoting both the accumulation of, and response to, stalled replication forks (33). Our group has recently shown that Ewing sarcoma cells are vulnerable to the loss of DDK activity, with its inhibition resulting in a failure to properly complete DNA replication, causing premature mitotic entry, mitotic catastrophe, and cell death (35). Interestingly, despite some evidence of premature mitotic entry, we found that the inhibition of DDK resulted in the phosphorylation of the mitotic regulator CDK1. CDK1 activity is regulated mainly by posttranslational modifications at tyrosine 14 and 15 (36). These phosphorylation events have been shown to negatively regulate CDK1 activity primarily through the prevention of nuclear translocation, delaying the onset of mitosis (37). While the regulation of CDK1 function at the mitotic border is rather complex, the main inhibitory phosphorylation is primarily mediated by WEE1 (38). This suggested to us that WEE1 is likely contributing to the mitotic entry delay upon DDK inhibition in Ewing cells, allowing for proper mitotic progression, potentially dampening the cytotoxic effects of DDK inhibitors (DDKi). Here, we show that the inhibition of WEE1 results in a potentiation of the negative effects of DDKis.

## Materials and Methods

### Cell Lines

A673 (Ewing; ATCC, catalog no. CRL-1598, RRID:CVCL_0080), RD-ES (Ewing; ATCC catalog no. HTB-166, RRID:CVCL_2169), and U2OS (non-Ewing, osteosarcoma; ATCC, catalog no. HTB-96, RRID:CVCL_0042) cell lines were obtained through ATCC. TC32 (Ewing; RRID:CVCL_7151) were obtained through Children's oncology group. A673 cells were cultured in DMEM, RDES, and TC32 in RPMI1640, U2OS in McCoys's 5A. All media was supplemented with 10% FBS and 1% antibiotic-antimycotic (Gibco Life Technologies). All cell lines were grown at 37°C and 5% CO_2_ and were used within 10 passages of receipt from ATCC or COG. Cells were tested for *Mycoplasma* most recently on March 15, 2022. All *Mycoplasma* testing was conducted by PCR through IDEXX BioAnalytics and was negative. Cell lines authentication was performed via short tandem repeat (STR) profiling. To acquire a DNA profile for each cell line, 15 STR loci and a gender-determining marker Amelogenin as well as positive and negative controls were amplified with the AmpFLSTR Identifiler Plus PCR Amplification Kit (Thermo Fisher Scientific, catalog no. A26182) on the Applied Biosystems Verti 96-well Thermal Cycler in 9600 Emulation Mode. The PCR products were run on the Applied Biosystems 3500 Genetic Analyzer and analyzed with Applied Biosystems GeneMapper v5. Eight of the 15 STRs and Amelogenin from the DNA profile for the cell line(s) are compared with ATCC STR database (https://www.atcc.org/STR%20Database.aspx?slp=1) and the DSMZ combined Online STR Matching Analysis (http://www.dsmz.de/fp/cgi-bin/str.html). All matches above 80% are considered to be from the same lineage.

### Chemical Inhibitors and Antibodies Used

Information for small molecular inhibitors and antibodies can be found in the materials excel sheet in the Supplementary Data section.

### Phospho-histone H3 FACS

Following indicated treatments outlined in figures or figure legends, cells were fixed in 4% paraformaldehyde and permeabilized with 0.5% Triton X-100 for 15 minutes at room temperature. Cells were then incubated with Alexa Fluor 488 anti-Histone H3 Phospho (Ser10) Antibody (5 μL/sample). Cells were then either placed in a propidium iodide (PI) solution or PBS and analyzed by flow cytometry. Total phosphor-histone H3–positive (pHH3^+^) cells were determined using the FCS Express software.

### Western Blotting

Following indicated treatments outlined in figures or figure legends, whole-cell lysates were collected and separated on an SDS-PAGE gel. Contents of gel were transferred to a polyvinylidenedifluoride membrane. Membranes were incubated in 5% milk in TRIS-buffered saline + 0.5% Tween-20 (TBST) for 1 hour to block prior to primary antibody incubation. Membranes were incubated with primary antibody dilutions (phospho-antibodies diluted in 5% BSA, in TBST non-phospho antibodies diluted in 5% milk in TBST). Membranes were incubated in primary antibody dilutions overnight at 4°C. The following day, membranes were washed in TBST and incubated in appropriate horseradish peroxidase–conjugate secondary antibodies for 1 hour. Protein abundances were detected using an Odyssey Western blot imaging system by LI-COR Biosciences.

### Cell Cycle

Following indicated treatments outlined in figures or figure legends, were fixed in ice-cold 70% ethanol at 4 degrees for 30 minutes. Total DNA content was stained using PI and analyzed via flow cytometry. DNA content and cell-cycle distributions were determined using the FCS Express software.

### Fluorescent Microscopy

Cells were cultured on glass coverslips and underwent the indicated treatments. Then cells were fixed using 4% paraformaldehyde and permeabilized using 0.5% Triton X-100. Total DNA content was stained using DAPI. Fluorescent images were captured on a Zeiss Axio Imager.A2 at 63× magnification.

### Annexin-V/PI Staining

Following indicated treatments outlined in figures or figure legends, cells were resuspended in Annexin-binding buffer. Apoptotic or dead cells were stained for Annexin-V and PI according to the manufacturer's instructions (Dead Cell Apoptosis Kit with Annexin V FITC and PI, for flow cytometry, Thermo Fisher Scientific, catalog no.: V13242). Cells were then analyzed via flow cytometry.

### Cell Viability

Cells were seeded at 5 × 10^3^ cells per well in a 96-well cell culture plate. A total of 24–48 hours after seeding, specified drugs concentrations were added to the appropriate well and cells were allowed to incubate with drug for 72 hours at 37 degrees. Cells were then incubated with 10 μL of cell counting kit-8 (WST-8/CCK8) reagent from GLPBIO for 1–3 hours. Absorbance was measured at 450 nm on a SpectraMax M2/M2E microplate reader (Molecular Devices). Final cell viability was determined relative to the average absorbance of solvent control-treated cells. For synergy calculations, CompuSyn software was used (39).

### Data Availability Statement

All of the data generated in this study are available within the article and its Supplementary Data files.

## Results

### DDK Inhibition Delays Mitotic Entry in Ewing Sarcoma Cells

**FIGURE 1:**
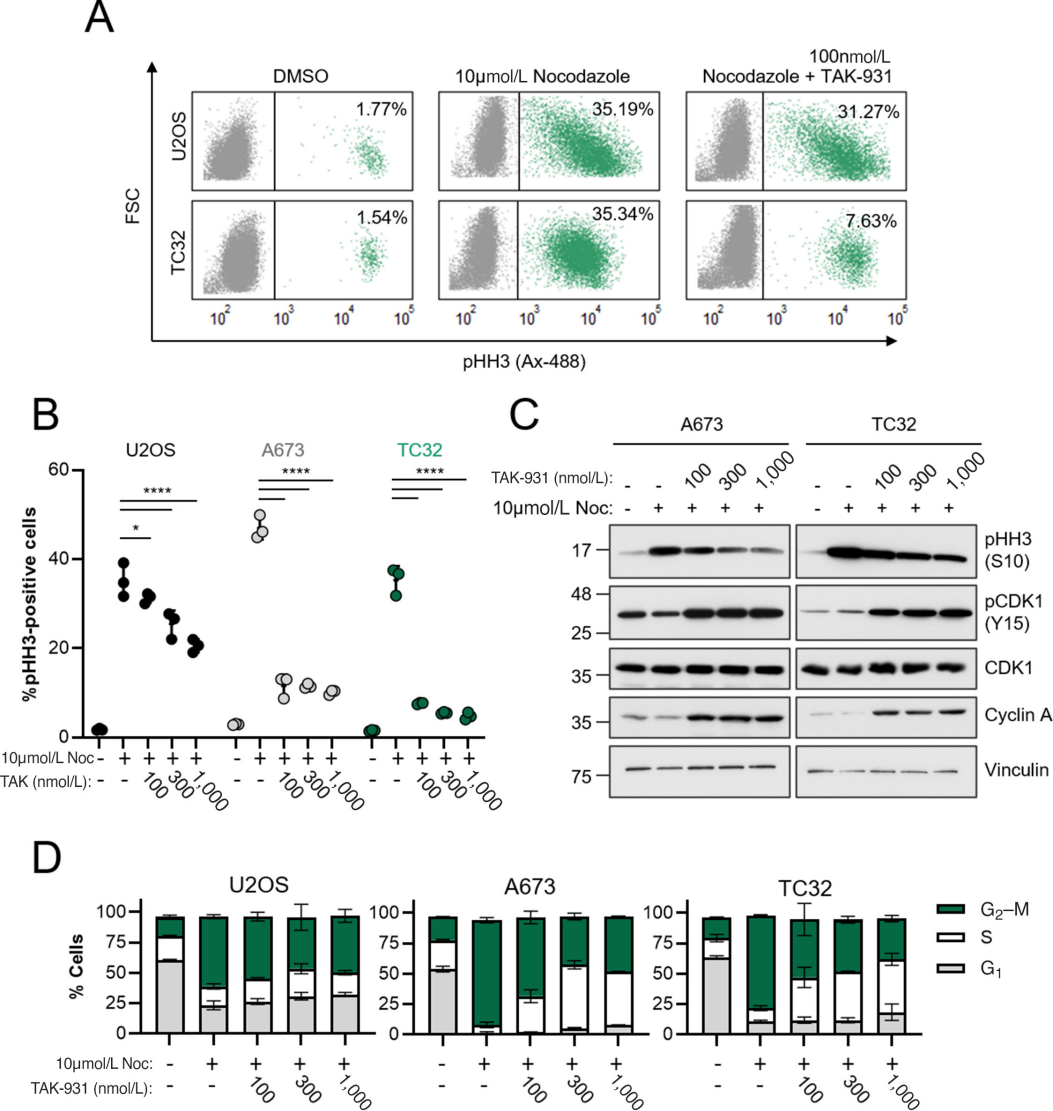
Inhibition of CDC7 (DDK) induces a delay in mitotic onset. Ewing sarcoma cells (A673 and TC32) as well as the non-Ewing osteosarcoma cell line U2OS were treated with either 0.1% DMSO, or 10 μmol/L nocodazole with increasing concentrations of TAK-931 (100 nmol/L, 300 nmol/L, and 1 μmol/L) for 24 hours. Cells were fixed and stained for pHH3 and pHH3^+^ cells were measured via FACS. **A,** Representative dot plots showing pHH3^+^ TC32 and U2OS cells upon nocodazole and TAK-931 treatment. **B,** Quantification of above-described experiment (*n* = 3 biological replicates, two-way ANOVA, *, *P* < 0.05; ****, *P* < 0.0001). **C,** A673 and TC32 cells were as described in **A**, protein lysates were collected, and Western blot analysis was performed for the specified proteins (Vinculin is used as a loading control). **D,** U2OS, A673 and TC32 cells were treated as described in **A**. Cells were then fixed and stained for DNA with propidium iodide and cell cycle was analyzed via FACS (*n* = 2 biological replicates).

DDK inhibition has previously been shown to significantly reduce DNA replication rates in Ewing sarcoma cells resulting in a prolongation of S-phase and a potential mitotic entry delay (35). To further investigate the extent of this mitotic entry delay, cells were cotreated with nocodazole (to induce a mitotic arrest) in combination with increasing concentrations of the DDKi TAK-931 (100 nmol/L–1 μmol/L) for 24 hours and the level of cells arrested in mitosis was measured via FACS and immunoblot. Nocodazole treatment was utilized here to allow for measurable differences in the relative level of mitotic cells. We found that DDK inhibition significantly reduced the level of nocodazole-induced mitotic cells in both the TC32 and A673 Ewing sarcoma cell lines as evidenced by the reduction in pHH3^+^ cells (Fig. 1A and B) as well as pHH3 protein levels (Fig. 1C). While this reduction was also observed in the osteosarcoma U2OS cells (used as a non-Ewing control line; Fig. 1B), it was much more significant in the Ewing sarcoma cells (A673 and TC32) with the lowest dose of TAK-931 tested (100 nmol/L), significantly reducing the level of nocodazole-induced mitosis (Fig. 1A–C). Importantly, the reduction in mitotic cells upon DDK inhibition was, seemingly, not due to a failure of the mitotic checkpoint as evidenced by the absence of an increase in cells in G_1_ (Fig. 1D). Instead, there appeared to be a gradual accumulation of cells with sub-4N DNA content (Fig. 1D; Supplementary Fig. S1) and increased pCDK1 and cyclin A levels (Fig. 1C) upon TAK-931 treatment, indicative of an extension of late-S–G_2_ and an inhibition of mitotic entry (36). Also, consistent with previous reports (35, 40, 41), we observed an increase in both the percentage of cells in late-S–G_2_–M and the level of phospho-CDK1 (Y15) in the Ewing sarcoma cells upon 8–24 hours treatment with two independent DDKis, TAK-931 and XL413 (Supplementary Fig. S2A–C), further suggesting that DDK inhibition induces a molecular inhibition of mitotic entry.

### WEE1 Activity is Required for the DDKi-induced Delay in Mitotic Entry in Ewing Sarcoma Cells

**FIGURE 2:**
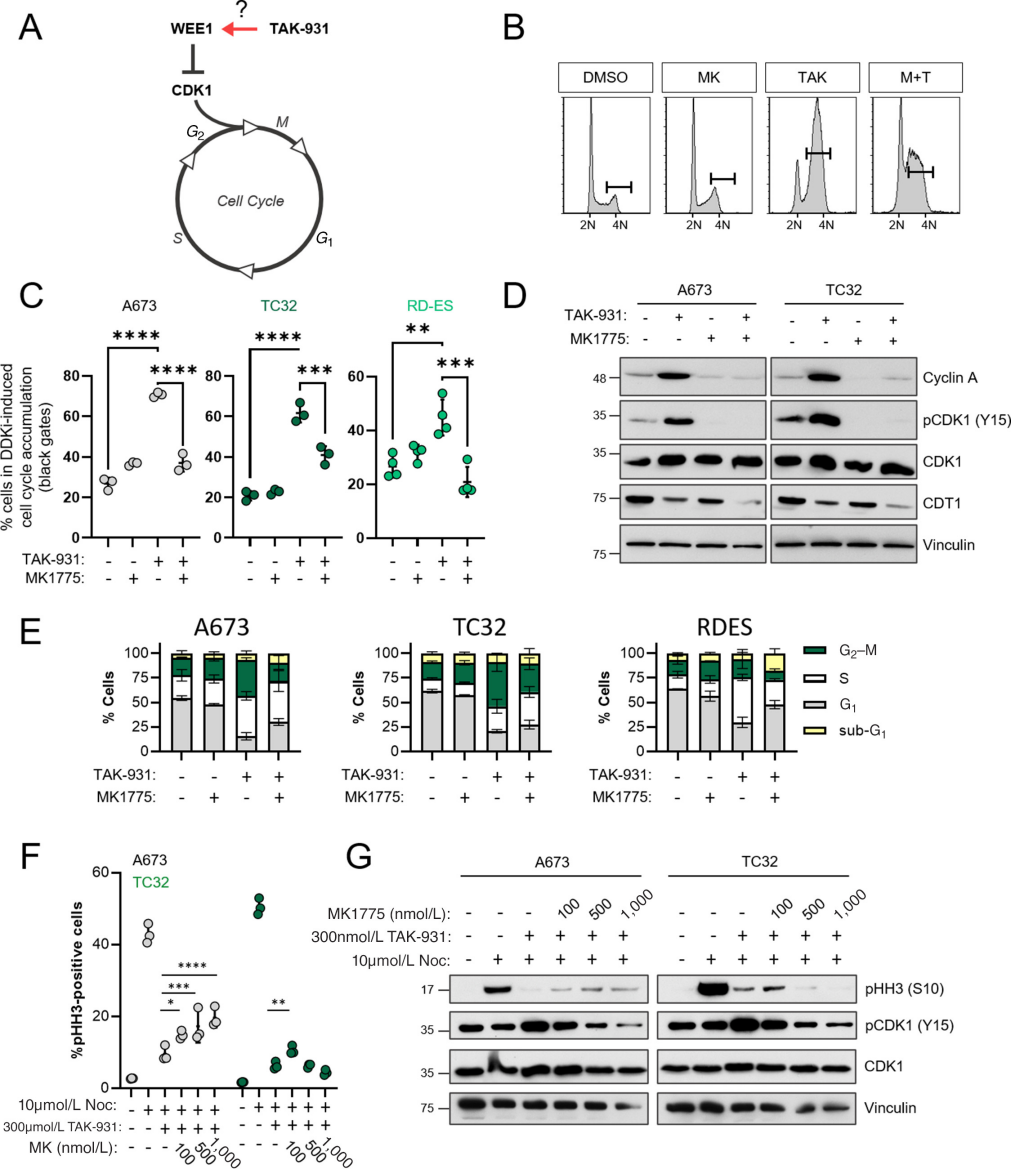
WEE1 activity is required for pCDK1 and mitotic entry delay upon DDK inhibition in Ewing cells. **A,** Simple schematic depicting WEE1’s role in the inhibition of mitotic onset. **B** and **C,** Cells were treated with 300 nmol/L TAK-931, 1 μmol/L MK1775 or combination for 24 hours. Cells were then fixed, and DNA was stained using PI. DNA content was analyzed via FACS. **B,** Representative histograms show a TAK-931 induced accumulation of cells during the cell cycle that is partially abrogated with the addition of MK1775 to TAK-931 (black gates). **C,** Quantification of DDKi-induced cell-cycle accumulation. Black gates from **B** were used to determine the proportion of these cells (*n* ≥ 3 biological replicates, one-way ANOVA, **, *P* < 0.005; ***, *P* < 0.001; ****, *P* < 0.0001). **D,** A673 and TC32 cells were treated with 300 nmol/L TAK-931 or 1 μmol/L MK1775 or the combination for 24 hours. Cell lysates were collected, and a Western blot analysis was performed for the specified proteins. Vinculin was used as the loading control. **E,** Cells were treated as described in **B** and **C**. Total DNA content was analyzed via FACS and cell-cycle distribution was determined (2N DNA content = G_1_, 4N DNA content = G_2_–M, >2N–<4N DNA content = S; *n* = 2 biological replicates). **F,** Cells were treated with 10 μmol/L nocodazole, 300 nmol/L TAK-931 with or without increasing concentrations of MK1775 (100 nmol/L, 500 nmol/L, and 1 μmol/L) for 24 hours. Cells were fixed and stained for pHH3 and analyzed via FACS (*n* = 3 biological replicates, two-way ANOVA, *, *P* > 0.05; **, *P* < 0.01; ***, *P* < 0.001; ****, *P* < 0.0001). **G,** Cells were treated as described in **F**. Whole-cell lysates were collected, and Western blot analysis was performed for the specified proteins. Vinculin was used as a loading control.

In the presence of DNA damage or underreplicated DNA, WEE1 works to inhibit mitotic entry by inhibiting CDK1 function through direct phosphorylation of tyrosine 15 (pCDK1-Y15; Fig. 2A; refs. 38, 42, 43). This allows for DNA repair and/or replication completion prior to mitotic entry, promoting faithful chromosomal segregation and cell division (43). Upon DDK inhibition, there is an emergence of a cell population within the cell cycle (Fig. 2B—black gates and C) that seems to represent an accumulation of cells in late-S–G_2_–M, potentially indicating a G_2_ arrest. This would be in line with previous reports (35). In accordance with WEE1’s role in the activation of the G_2_ checkpoint, WEE1 inhibition (1 μmol/L MK1775) was sufficient to reduce this population (Fig. 2B and C). Also, WEE1 inhibition reduced the DDKi-induced increase in pCDK1 (Y15) and cyclin A levels (Fig. 2D), further suggesting that what we are observing is a WEE1-dependent DDKi-induced G_2_ checkpoint activation. Importantly, the reduction in the proportion of cells represented by the black gates in Fig. 2B and C was accompanied by a slight increase in the proportion of cells in G_1_ (Fig. 2E—gray bars), suggesting a potential G_2_–M checkpoint bypass and mitotic exit. Interestingly, however, there did not appear to be an increase in the abundance of the licensing factor CDT1 (Fig. 2B), whose protein stability is mainly limited to G_1_ (44). To further investigate the extent of this apparent WEE1-mediated G_2_ checkpoint activation and mitotic entry delay, Ewing sarcoma cells were cotreated with nocodazole and the DDKi TAK-931 with or without increasing concentrations of MK1775 (100 nmol/L–1 μmol/L) for 24 hours and total mitotic cells were measured via FACS and immunoblot. We found that, while TAK-931 treatment reduced the level of pHH3 that is induced by nocodazole, the addition of MK1775 partially restored pHH3 levels in a dose-dependent manner (Fig. 2F and G). This effect was more pronounced in the A673 cells while only the lowest dose of MK1775 was able to, slightly but consistently, restore the level of pHH3^+^ cells in the TC32 cells. However, there was a dose-dependent reduction in p-CDK1(Y15) upon MK1775 treatment in both Ewing cell lines (Fig. 2G), indicating that the phosphorylation of CDK1 upon DDK inhibition is dependent on WEE1. These results suggest that WEE1 activity contributes to the phosphorylation of CDK1, G_2_ arrest, and mitotic entry delay upon DDK inhibition in Ewing sarcoma cells.

### Combined Inhibition of WEE1 and DDK Causes Premature Mitotic Entry and Mitotic Abnormalities in Ewing Sarcoma Cells

**FIGURE 3:**
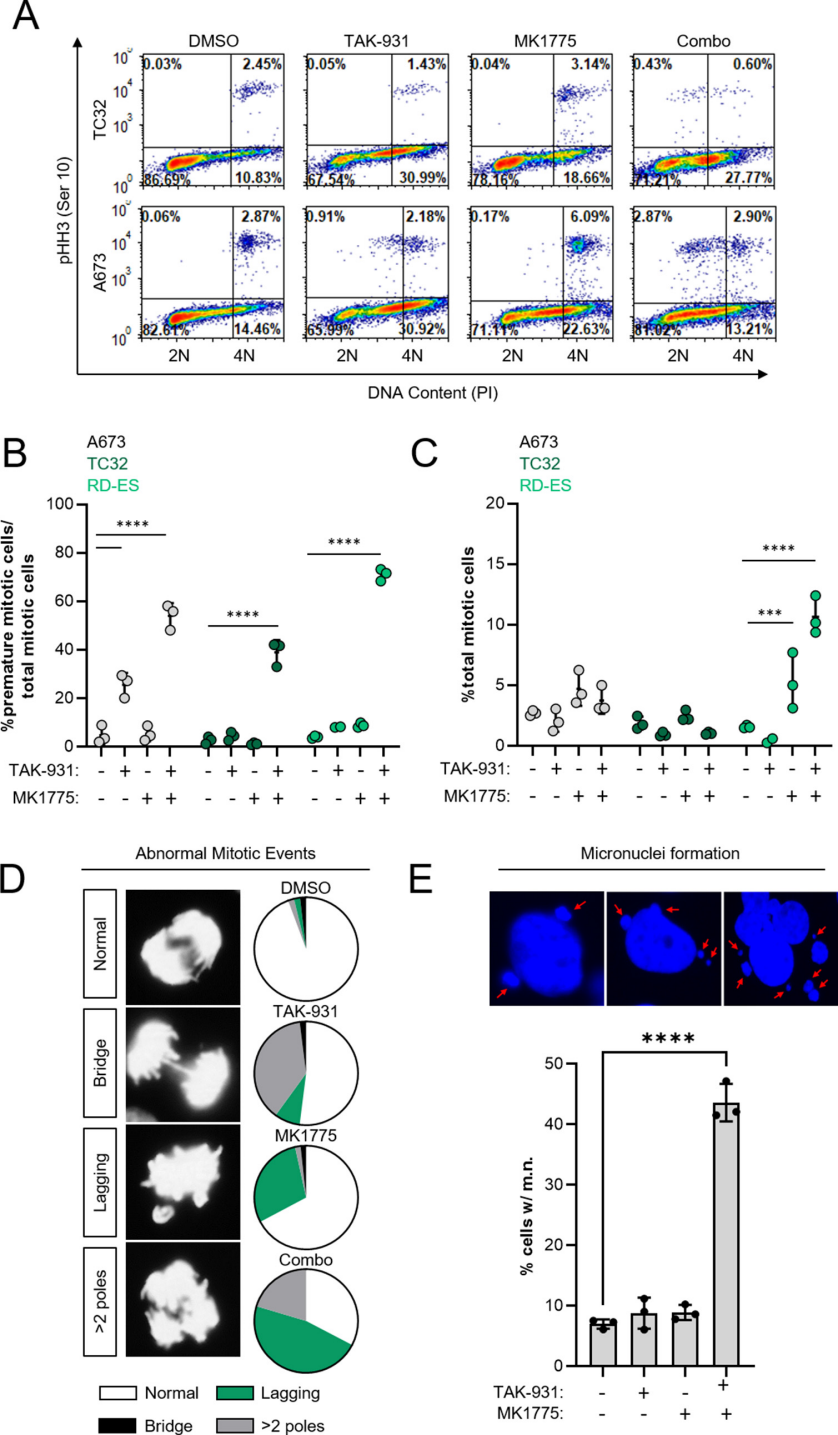
Combined inhibition of WEE1 and DDK induces premature mitotic entry, mitotic progression abnormalities and mitotic catastrophe in Ewing cells. **A**–**C,** Cells were treated with 300 nmol/L TAK-931, 1 μmol/L MK1775 or combination for 24 hours. Cells were then fixed and stained for DNA content (PI) and pHH3 and analyzed via FACS. Premature mitotic cells were identified as cells that stained positive for pHH3 that contain <4N DNA content (top left quadrant). **A,** Representative dot plots. **B,** Quantification of percentage of premature mitotic cells as a proportion of total mitotic cells [(premature mitotic cells/total mitotic cells) * 100%] (*n* = 3 biological replicates, two-way ANOVA, ****, *P* < 0.0001). **C,** Quantification of total mitotic cells (pHH3^+^ cells; *n* = 3 biological replicates, two-way ANOVA, ****, *P* < 0.0001). **D,** A673 cells were treated with 300 nmol/L TAK-931, 1 μmol/L MK1775 or combination for 24 hours. DNA was stained using DAPI and abnormal anaphase events were quantified (*n* = 2 biological replicates, ≥50 anaphase events analyzed per condition, per replicate). **E,** A673 cells were treated as described in **D**. Total DNA was stained using DAPI and micronucleated cells were quantified (*n* = 3 biological replicates, ≥ 100 cells were analyzed per condition, per replicate, one-way ANOVA, ****, *P* < 0.0001).

WEE1 activity prevents the mitotic entry of cells with damaged or underreplicated DNA (37, 38, 42, 45). Because DDK inhibition significantly reduces the replicative capacity of Ewing cells and prolongs S-phase, and there is no evidence to suggest an acute induction of DNA damage upon DDKi treatment (35), we hypothesized that WEE1 activity is likely preventing mitotic entry due to the presence of underreplicated chromosomes. We reasoned then, that the combined inhibition of WEE1 and DDK would result in the unscheduled mitotic entry of Ewing cells with underreplicated DNA (37). To investigate this, cells were cotreated with TAK-931 and MK1775 (T+M) for 24 hours followed by analysis of DNA content and pHH3 levels via FACS. We found that the combination of T+M resulted in a significant level of pHH3^+^ cells with sub-4N DNA content (Fig. 3A—top left quadrant), indicative of premature mitotic entry, in all Ewing lines tested (Fig. 3A and B). Consistent with previous reports (35), TAK-931 treatment alone was able to induce premature mitotic entry in the A673 cells (Fig. 3A—bottom and B—white dots). Interestingly, this was not observed in TC32 and RDES cells. Importantly, however, the combination treatment induced premature mitotic entry in all Ewing cell lines to similar levels, indicating that the mechanism inhibiting DDKi-induced premature mitotic entry in the TC32 and RDES cells can be overcome by the addition of a WEE1 inhibitor. Interestingly, there was only a slight increase in total mitotic cells in the A673 and RDES cells and no apparent increase in the TC32 cells upon T+M treatment (Fig. 3C), indicating that these cells may still be progressing through and exiting mitosis.

Mitotic entry delay is a crucial component of the G_2_ checkpoint (36). Checkpoint failure can result in unscheduled mitotic progression resulting in the formation of aberrant mitotic structures and subsequent mitotic catastrophe (37, 43). WEE1-mediated inhibition of CDK1 resides at the center of this checkpoint (38). The large amount of premature mitotic entry induced by T+M treatment prompted us to investigate its potential to cause abnormal mitotic progression in Ewing cells. Immunofluorescent imaging of chromosomal DNA (using DAPI) revealed a significant increase in the frequency of mitotic abnormalities induced by the combination treatment, specifically mitotic events with anaphase bridges, apparent lagging chromosomes and cells with >2 divisional poles (Fig. 3D). Interestingly, the most common phenotype observed was cells with broken or lagging chromosomes, either chromosome fragments or entire chromosomes (Fig. 3D—green area). We also observed a reduction in the number of cells that had >2 poles when comparing TAK-931 treatment alone to T+M (Fig. 3D—gray area). This is in line with the idea that a significant mitotic entry delay is required to overduplicate centrosomes and cause cells to divide in an asymmetric manner (41, 46). Furthermore, there was a significant increase in the proportion of cells harboring micronuclei upon T+M treatment, indicative of mitotic catastrophe (Fig. 3E). Our group has previously shown that DDK inhibition alone can induce mitotic catastrophe in Ewing sarcoma cells, but this phenomenon only occurs after prolonged treatments (∼48 hours; ref. 35). Here, we are observing clear signs of mitotic catastrophe upon treatment with T+M at 24 hours, suggesting that the addition of a WEE1 inhibitor can exacerbate these defects and elicit these phenotypes at earlier timepoints. Taken together, these results demonstrate that the combined inhibition of DDK and WEE1 causes premature mitotic entry, mitotic progression abnormalities and ultimately mitotic catastrophe in Ewing sarcoma cells.

### DDK and WEE1 Inhibitors Synergize to Kill Ewing Sarcoma Cells *In Vitro*

**FIGURE 4:**
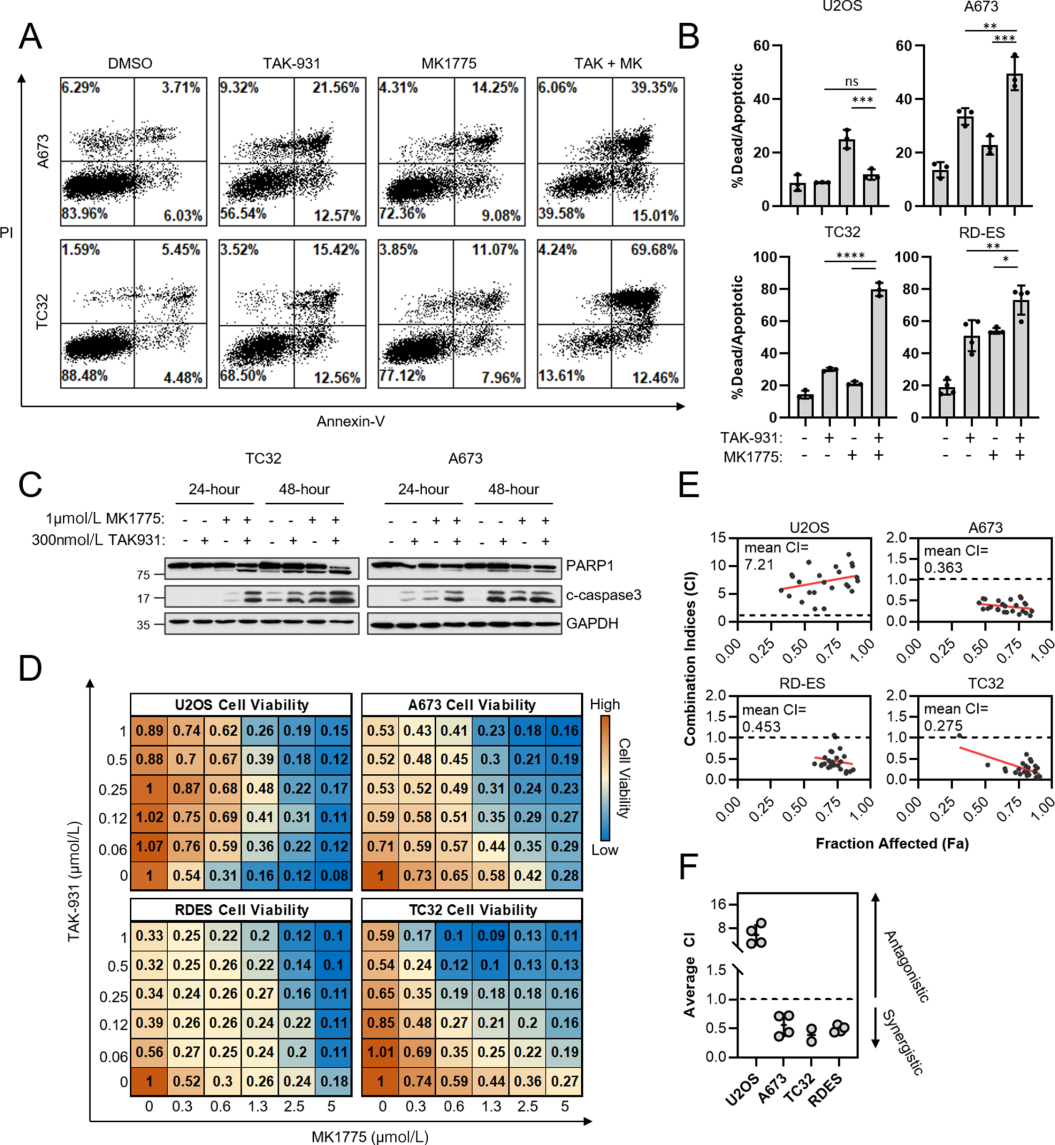
DDK and WEE1 inhibitors synergistically reduce Ewing sarcoma cell viability. **A** and **B,** Cells were treated with 300 nmol/L TAK-931, 1 μmol/L MK1775 or combination for 48 hours. Cells were then stained for live or dead with PI and apoptosis with Annexin-V. **A,** Representative dot plots. **B,** Quantification of dead or apoptotic cells of the above-described experiment (*n* ≥ 3 biological replicates, one-way ANOVA, *, *P* > 0.05; **, *P* < 0.01; ***, *P* < 0.001; ****, *P* < 0.0001). **C,** A673 and TC32 cells were treated as described in **A**. Whole-cell lysates were collected, and Western blot analysis was performed for the following proteins. GAPDH was used as a loading control. **D,** Cells were treated with increasing combinations of TAK-931 (1–0.0625 μmol/L) and MK1775 (5–0.125 μmol/L) for 72 hours and cell viability was assessed. **C,** Representative cell viability grid. Orange represents areas of high viability while blue represents areas of low viability. Representative results of four biological replicates. **E,** Cell viability results from **D** were used to calculate CI values with the CompuSyn software (referenced in text). CI < 1 = synergistic, CI of 1 = additive, CI > 1 = antagonistic. Fraction affected is calculated by subtracting the cell viability from 1 (100% cell viability would have a fraction affected of 0, while 0% cell viability would have a fraction affected of 1). Red line represents slope of the data. A negative slope indicates that, as the fraction affected becomes larger, the CI values approach. This means that the combinations that display the highest level of synergy also display the highest level of cell killing; this is desirable. Representative results of four biological replicates. **F,** Average CI values from **E** (*n* = 4 biological replicates; TC32 = 2 biological replicates).

A common outcome of mitotic catastrophe is cell death, typically through apoptosis (47). Because of the significant degree of mitotic entry abnormalities and mitotic catastrophe induced by T+M, we were interested in determining the cytotoxic effects of this combination treatment. We found that T+M-induced Annexin-V/PI staining (indicative of cell death and apoptosis) was significantly greater than the level of that induced by either drug alone in all Ewing lines tested (Fig. 4A and B). These results were confirmed via immunoblot (Fig. 4C). Cleaved-PARP and cleaved-caspase 3 levels upon T+M treatment were noticeably higher than either drug alone at both 24 and 48 hours in both the A673 and TC32 cells (Fig. 4C), suggesting a possible synergistic relationship. Importantly, this combination displayed limited cytotoxic effects in the U2OS cells, highlighting the unique response within the Ewing cell lines (Fig. 4B). In fact, in the U2OS osteosarcoma cells, the inhibition of DDK reduced the level of apoptosis induced by the WEE1 inhibitor alone (Fig. 4B), suggesting a potential antagonistic relationship within these cells.

On the basis of our above findings and the proposed mechanism of action of T+M, we hypothesized that this combination would display a synergistically cytotoxic relationship within Ewing sarcoma cells. To test this, cells were treated with several combinations of TAK-931 and MK1775 (25 combinations in total) for 72 hours and cell viability was assessed. We found that, in line with previous reports (35), Ewing sarcoma cells were sensitive to TAK-931 alone (Fig. 4D). Importantly, when increasing concentrations of MK1775 were added, a greater loss of viability was observed (Fig. 4D). To assess synergy, combination index (CI) values were calculated using the CompuSyn software (39). This allows for a mathematical evaluation of drug relationships with regards to cellular effects, in this case, cell viability. A CI value of less than 1 indicates that, at the specified concentrations, the two compounds display synergism. Anything above 1 is considered to be antagonistic and a CI value of 1 is deemed additive. Within the Ewing sarcoma cells, (almost) all combinations tested had a CI value less than 1 (Fig. 4E). Importantly, the average CI value for all 25 combinations was below 1 (A673: 0.557; TC32: 0.384; RDES: 0.4915; Fig. 4F) in all Ewing lines tested, indicating that this combination does in fact synergistically reduce viability of Ewing sarcoma cells. Furthermore, in line with the results in Fig. 3B, this combination was antagonistic in the U2OS osteosarcoma cells (average CI: 5.798; Fig. 4D–F), again emphasizing the unique response within Ewing cells.

### T+M-induced Apoptosis Requires Mitotic Progression

**FIGURE 5:**
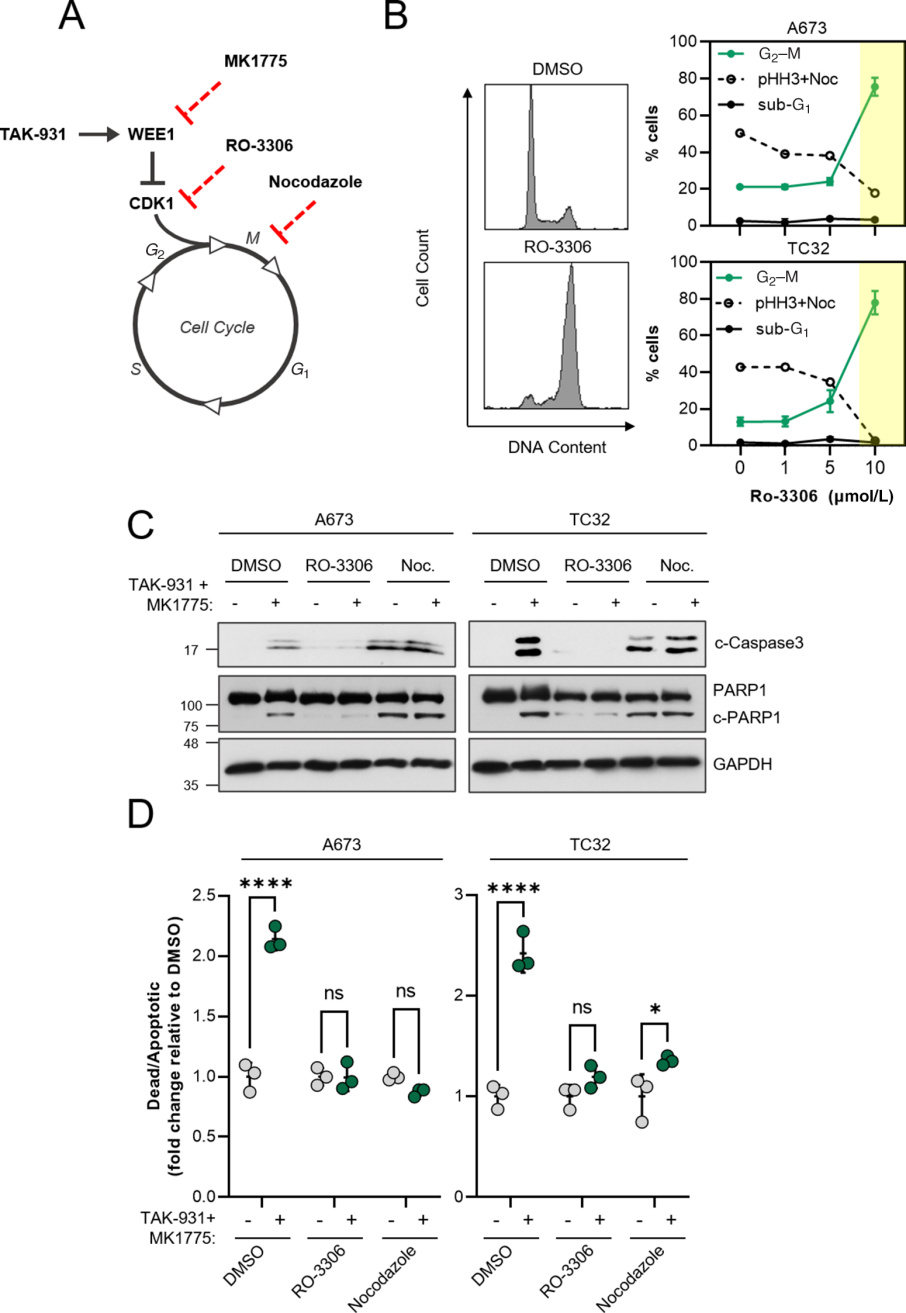
Apoptotic induction of TAK-931 + MK1775 requires mitotic entry or progression. **A,** Schematic representing the targets of the various drugs used in this experiment. Black arrow depicts the findings from Fig. 2—TAK-931 activates WEE1. **B,** Cells were treated with 10 μmol/L nocodazole in combination with increasing concentrations of RO-3306. Cells were then fixed and stained for DNA content and pHH3 (*n* = 2 biological replicates). **C,** Cells were treated with 300 nmol/L TAK-931 in combination with 1 μmol/L MK1775 with or without 10 μmol/L RO-3306 or 10 μmol/L nocodazole for 24 hours. Whole-cell lysates were collected, and Western blot analysis was performed for the specified proteins. GAPDH was used a loading control. **D,** Cells were treated as described in **C**. Cells were then stained for live or dead with PI and apoptosis with Annexin-V. Quantification of dead or apoptotic cells is shown. Relative dead or apoptotic cells were calculated on the basis of the level of dead or apoptotic of cells that were not given the T + M combination (e.g., T + M + RO-3306 is relative to RO-3306 alone; *n* = 3 biological replicates, two-way ANOVA, *, *P* < 0.05; ****, *P* < 0.0001).

The T+M-induced apoptotic induction is likely a result of premature mitotic entry and aberrant mitotic progression (Fig. 3; ref. 35). Progression through mitosis with underreplicated or damaged DNA has been shown to result in mitotic catastrophe-associated DNA damage and cell death (48). Forced inhibition of mitosis with the use of CDK1 inhibitors (RO-3306) or microtubule poisons (nocodazole) is sufficient to hinder the entry or progression of mitosis (Fig. 5A) resulting in a cell-cycle arrest in G_2_ (Fig. 5B; Supplementary Fig. S3) and a temporary hindrance of the effects of errors that result from abnormal chromosomal segregation (49).

To determine the contribution of mitotic progression to T+M-induced apoptosis, cells were cotreated with T+M in combination with either RO-3306 or nocodazole for 24 hours and apoptotic induction was assessed via immunoblot. We found that, while T+M induced a significant level of cleaved-PARP and cleaved-caspase 3, indicative of apoptosis, there was no significant induction when cells were cotreated with RO-3306 or nocodazole (Fig. 5C). Importantly, while RO-3306 and nocodazole treatment alone induce some apoptosis, there was no significant increase when T+M was added (Fig. 5C). These results were confirmed using Annexin-V/PI staining (Fig. 5D). We found that, again, T+M treatment did not induce a significant level of apoptosis above that of the RO-3306 or nocodazole alone, suggesting that CDK1 inhibition or microtubule polymerization inhibition can suppress the apoptotic effects of T+M. Together, these results suggest that mitotic progression is required to elicit the cytotoxic effects induced upon combination treatment with DDK and WEE1 inhibitors in Ewing sarcoma.

## Discussion

Treatment of Ewing sarcoma has remained largely consistent for decades despite the clear evidence of treatment toxicity and long-term negative side effects (6). There has been a growing need for the development of novel, less toxic therapeutics that can effectively treat the disease while limiting toxicities. RS offers a potential therapeutic entry point within Ewing tumors (14). Here, we have discovered that the inhibition of WEE1 can be used to enhance the sensitivity of Ewing sarcoma cells to DDKis. Specifically, the addition of a WEE1 inhibitor forces DDKi-treated Ewing sarcoma cells into mitosis prematurely. This results in the mitotic entry of cells with underreplicated DNA causing elevated levels of abnormal mitoses, mitotic catastrophe, and a synergistic reduction in cell viability. This is the first study to utilize the inhibition of DDK in combination with WEE1, offering a unique therapeutic combination that may prove efficacious for Ewing sarcoma as well as a variety of other tumor types that have a similar sensitivity to DDKis. Additional studies are needed to (i) identify vulnerable cancer subtypes and (ii) develop biomarkers that are indicative of therapeutic response.

WEE1 function resides at the border of mitosis, acting as a gatekeep of sorts for mitotic entry (38, 45). The G_2_–M checkpoint operates in, what some have termed, a “node-like manner,” where the central node is the phosphorylation and deactivation of CDK1 (36). There are two main events that control CDK1 phosphorylation. The first is dependent upon WEE1, which promotes CDK1 phosphorylation and mitotic entry inhibition. The second is dependent upon the phosphatase CDC25C, which dephosphorylates CDK1 and promotes mitotic onset. Importantly, the protein stability of CDC25C is negatively regulated by CHK1 phosphorylation, which targets it for degradation via the proteosome (50). Therefore, along with WEE1, CHK1 plays a large role in the enforcement of the G_2_ checkpoint and would be a prime candidate to be inhibited in combination with DDKis. However, the decision to focus on the inhibition of WEE1 to overcome the DDKi-induced mitotic entry delay in Ewing sarcoma was based on the following three things. (i) CHK1 activity negatively regulates the removal of the WEE1-mediated phosphorylation of CDK1 through its regulation of CDC25C (50). The inhibition of WEE1 bypasses CHK1’s role in this process, as CDK1 phosphorylation is dependent on WEE1, deeming the phosphatase, CDC25C, less necessary for CDK1 function. (ii) In our previous study (35), in which we show that Ewing cells are sensitive to DDKis, we found some, but minimal, ATR-mediated activation of CHK1, suggesting a less than robust level of CHK1 activity upon DDK inhibition. (iii) Ewing sarcoma cells have been shown to be relatively resistant to CHK1 inhibitors as single agents, possibly due to an overabundance of inactive CHK1 protein (51). For these reasons, the inhibition of WEE1 seems to present the more viable approach. Also, compared with CHK1 inhibitors, WEE1 inhibitors have been met with greater success in clinical use (52).

WEE1 inhibitors have been used to force the premature mitotic entry of cells arrested in G_2_ (38) upon treatment with DNA-damaging agents such as platinum agents [cisplatin (53)] and radiotherapy (54). These combinations prove to be especially efficacious in tumor cells harboring mutant or deleted TP53 (55). The reason being, is that cells with DNA damage in G_1_ rely on the function of the p53-p21 axis to induce a cell-cycle arrest and hinder S-phase entry. In the absence of functional p53, cells enter S-phase and accumulate more DNA damage before reaching G_2_–M. Then, WEE1 function is relied upon to inhibit the mitotic entry of cells harboring DNA damage. WEE1 inhibition, allows for mitotic entry and progression of cells with DNA damage, resulting in mitotic failure and cell death. The use of DDKis in place of traditional chemotherapy differs from this classical approach in many ways, mainly, DDK inhibition does not induce DNA damage and does not rely on a mutant p53 for cell-cycle progression. This unique combination may also eliminate the need to include any form of traditional chemotherapy or DNA damage–inducing agent, potentially limiting systemic toxicities. Future *in vivo* studies focused on the use of DDK and Wee1 inhibitors both in combination with cytotoxic chemotherapy, and as a way to reduce or eliminate the need for chemotherapy are planned.

While we have displayed this combination's potential for the treatment of Ewing sarcoma, we do not believe its therapeutic efficacy is limited to just these tumors. Ewing sarcoma provides an advantageous model given the response to DDKi treatment. DDK activity is clearly important for the maintenance of replicative capacity and faithful transition into mitosis in Ewing cells, as evidenced by the reduction to replication rates and significant mitotic entry delay upon its inhibition. This leaves the cells reliant on the G_2_ checkpoint for accurate monitoring of DNA replication completion prior to mitotic entry, leaving them vulnerable to the inhibition of this process. This was the basis for our hypothesis that drove the above-described work. According to this logic, the sensitivity to DDKi + WEE1i relies on the initial sensitivity to DDK inhibition alone. This means then, theoretically, any tumor type that would respond to DDK inhibition in a similar manner to Ewing cells, will display comparable sensitivity to DDKi + WEE1i. However, the underlying mechanism of DDKi sensitivity within Ewing cells remains elusive, though it is not a complete mystery.

DDK plays a primary role in the activation of replication origins by phosphorylating MCM and recruiting CDC45 and the GINS complex (30). Many licensed origins are not fired during a typical S-phase. Instead, they are reserved for activation in times of need (56–58). DDK activity is required for this process (33, 34). This, along with our current understanding of Ewing sarcoma biology and the Ewing sarcoma cell response to DDKis (35) has led us to hypothesize that an increased reliance on dormant origin usage due to the presence of high levels of fork stalling events leaves the cells dependent upon DDK. This is based on the fact that there is substantial evidence to support the sensitivity to RSR kinase inhibitors (59–61), highlighting the presence of intrinsic RS, the source of which may be transcription-associated DNA secondary structures (27). Also, Ewing cells are known to display sensitivity to alterations in nucleotide levels (59, 62), suggesting high dNTP usage or lack of production. Given also that Ewing cell lines display reduced fork velocity rates, a phenomenon that can be caused by elevated origin usage, it is feasible to imagine the presence of a phenotype within these tumors which utilizes elevated replication origin activity to compensate for an increased propensity of fork stalling. Therefore, we hypothesize that tumors with known origin activity alterations or a reliance on dormant origin usage are prime candidates for DDK + WEE1 inhibition. Work by others suggests that some types of pancreatic, esophageal, ovarian, and breast cancers may be vulnerable to DDKi (63), especially in combination with chemotherapeutic agents. This study did not look at potential synergy with Wee1i or utilize Ewing sarcoma models. Additional future studies based on our hypothesis using large-scale cell line–based screens are warranted, as is an effort to identify biomarkers predictive of response.

Importantly, both TAK-931 and MK1775 are orally bioavailable and have been studied in human patients with both drugs being tested as single agents in multiple clinical trials for various tumor types (clincaltrials.gov). TAK-931 was first shown to have an acceptable safety profile and early signs of clinical antitumor activity in a first-in-human phase I trial in patients with advanced solid tumors. Isolated neutropenia was the dose-limiting toxicity with the tested schedule, and there was good pharmacodynamic evidence of target engagement (Clinical trial information: NCT02699749). The Wee1 inhibitor MK1775/AZD1775 has been and/or is currently being tested in dozens of human tumors (clinicaltrials.gov), though not in Ewing sarcoma to date. To our knowledge, the combination of DDKi + Wee1i has not been tested in humans to date. Our group is currently initiating a large-scale preclinical study to investigate the efficacy of this combination *in vivo* and to ensure minimal toxicity with the ultimate goal of introducing this combination as a potentially therapeutic opportunity for human patients.

## Supplementary Material

Materials Spreadsheet MS1 This table provides additional information about the materials used in this study.

Figure S1 This figure includes supplementary data to show that CDC7 (DDK) inhibition delays mitotic entry in Ewing cells.

Figure S2 This figure includes supplementary data that shows that CDC7 (DDK) inhibition increases pCDK1 (Y15) and causes a cell cycle accumulation in Ewing cells.

Figure S3 This figure includes supplementary data that shows CDK1 inhibition or nocodazole treatment inhibits mitotic entry/progression in Ewing cells.
