# Supplementary figures and images for: WEE1 Inhibition Augments CDC7 (DDK) Inhibitor–induced Cell Death in Ewing Sarcoma by Forcing Premature Mitotic Entry and Mitotic Catastrophe
Source: Cancer Res Commun. 2022 Jun 20;2(6):471–82. doi: 10.1158/2767-9764.CRC-22-0130 (PMC9635308; doi:10.1158/2767-9764.CRC-22-0130)

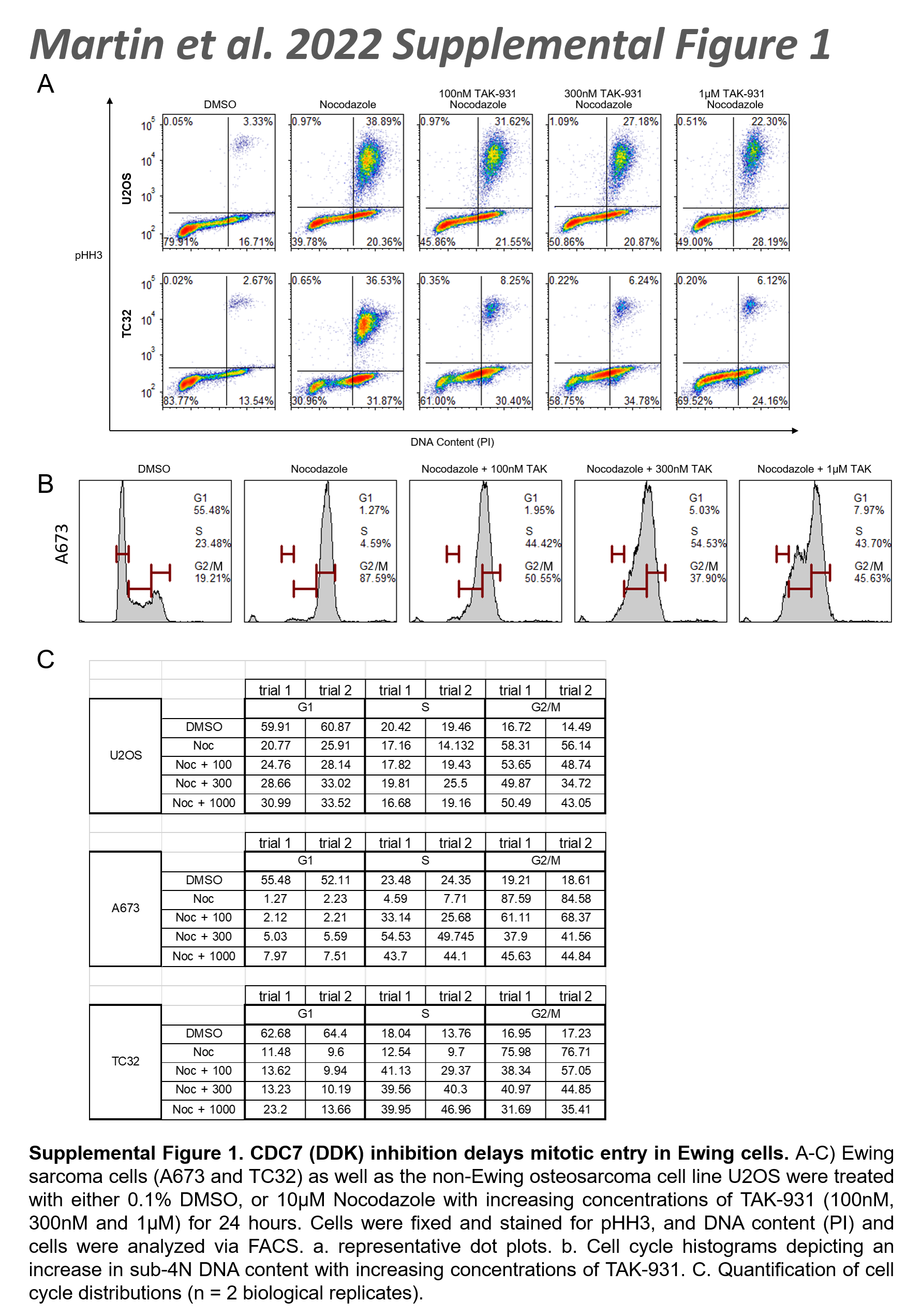

Supplement: Figure S1 — This figure includes supplementary data to show that CDC7 (DDK) inhibition delays mitotic entry in Ewing cells. [file crc-22-0130-s02.png]

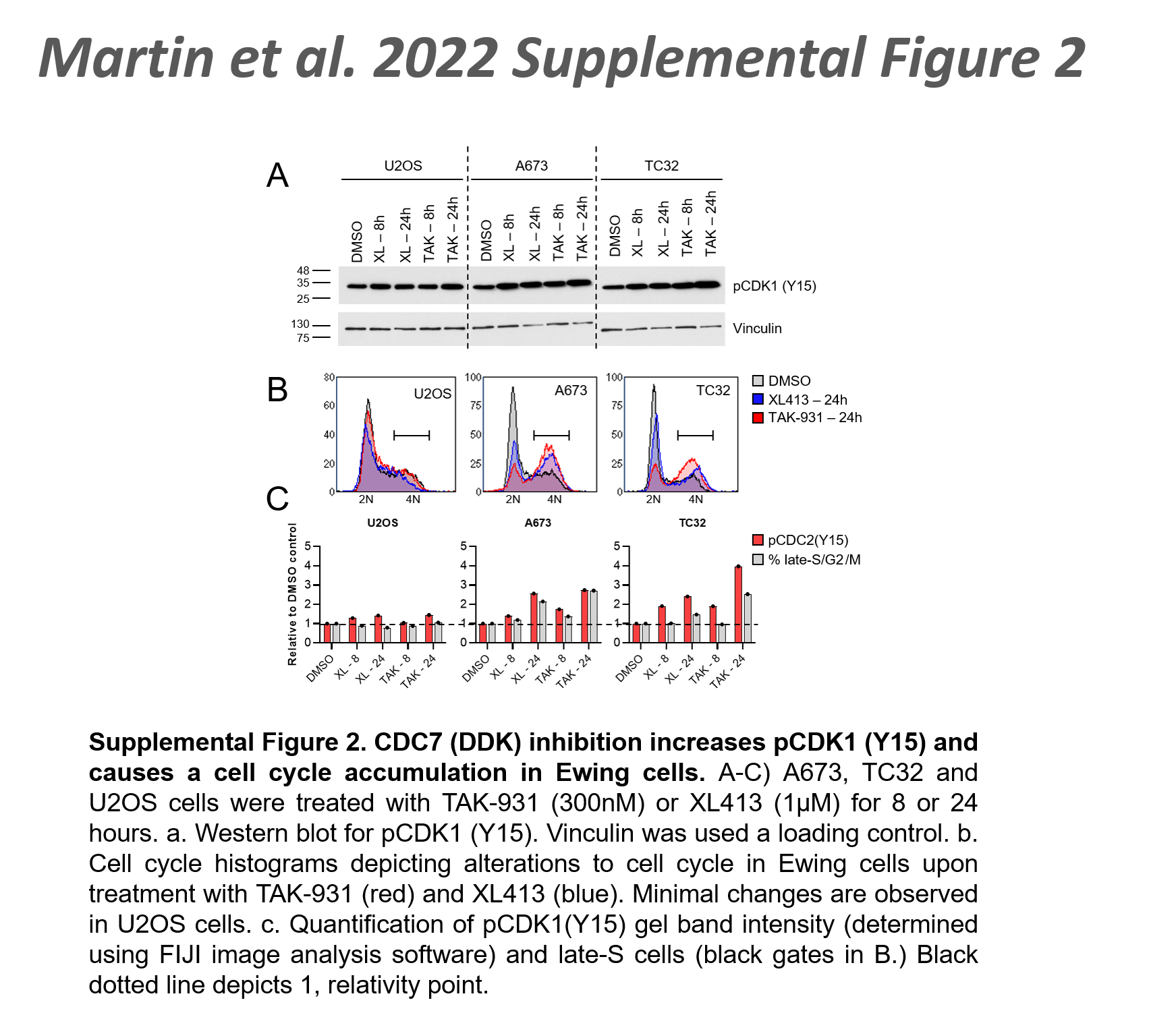

Supplement: Figure S2 — This figure includes supplementary data that shows that CDC7 (DDK) inhibition increases pCDK1 (Y15) and causes a cell cycle accumulation in Ewing cells. [file crc-22-0130-s03.png]

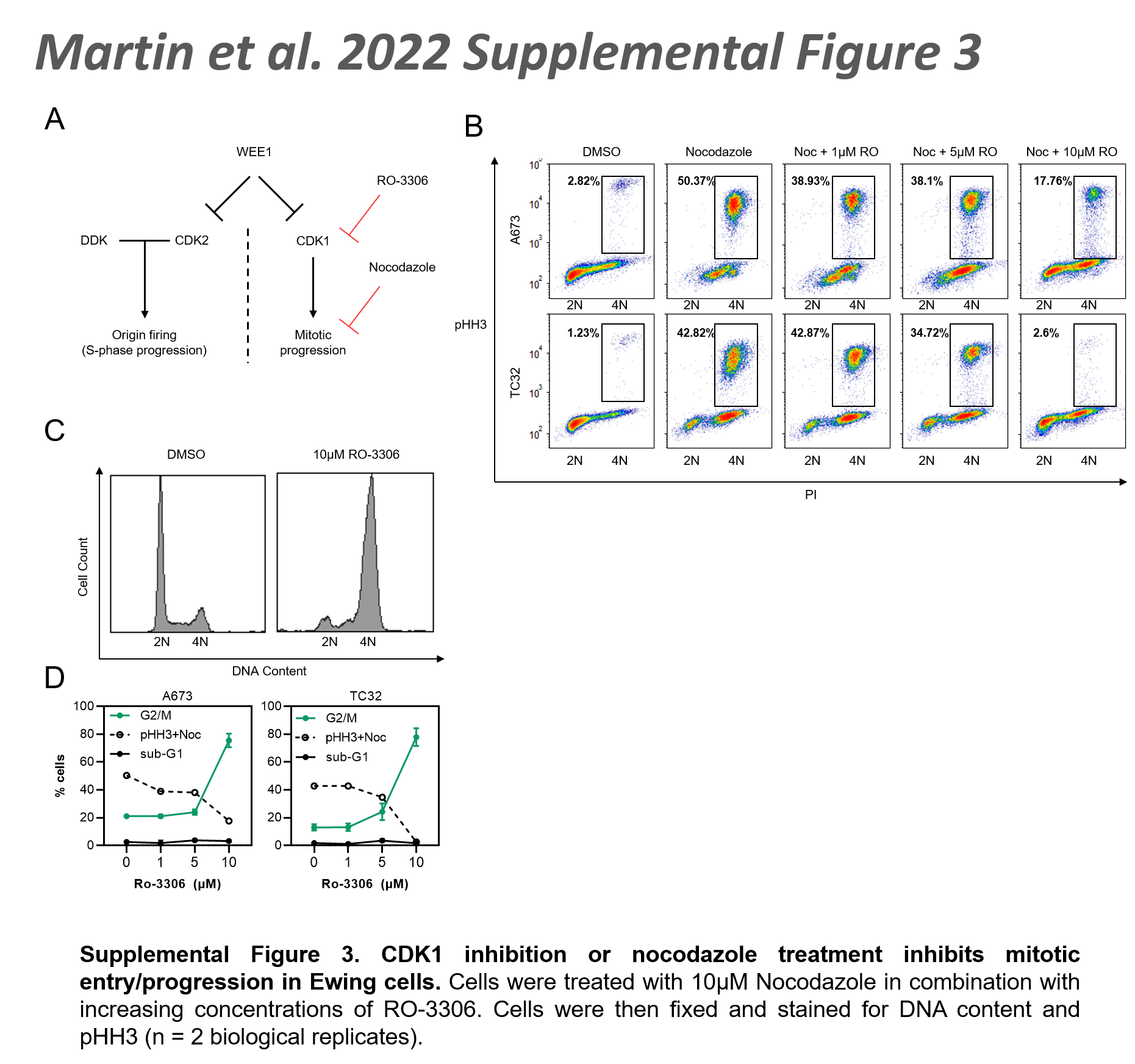

Supplement: Figure S3 — This figure includes supplementary data that shows CDK1 inhibition or nocodazole treatment inhibits mitotic entry/progression in Ewing cells. [file crc-22-0130-s04.png]
